# Genome-wide association study of response to cognitive–behavioural therapy in children with anxiety disorders

**DOI:** 10.1192/bjp.bp.115.168229

**Published:** 2016-09

**Authors:** Jonathan R. I. Coleman, Kathryn J. Lester, Robert Keers, Susanna Roberts, Charles Curtis, Kristian Arendt, Susan Bögels, Peter Cooper, Cathy Creswell, Tim Dalgleish, Catharina A. Hartman, Einar R. Heiervang, Katrin Hötzel, Jennifer L. Hudson, Tina In-Albon, Kristen Lavallee, Heidi J. Lyneham, Carla E. Marin, Richard Meiser-Stedman, Talia Morris, Maaike H. Nauta, Ronald M. Rapee, Silvia Schneider, Sophie C. Schneider, Wendy K. Silverman, Mikael Thastum, Kerstin Thirlwall, Polly Waite, Gro Janne Wergeland, Gerome Breen, Thalia C. Eley

**Affiliations:** Jonathan R. I. Coleman, MSc, King's College London, Institute of Psychiatry, Psychology and Neuroscience (IoPPN), MRC Social, Genetic and Developmental Psychiatry (SGDP) Centre, UK; **Kathryn J. Lester**, DPhil, King's College London, IoPPN, MRC SGDP Centre, UK, and School of Psychology, University of Sussex, UK; **Robert Keers**, PhD, **Susanna Roberts**, MSc, King's College London, IoPPN, MRC SGDP Centre, UK; **Charles Curtis**, MSc, King's College London, IoPPN, MRC SGDP Centre, UK, and National Institute for Health Research Biomedical Research Centre, South London and Maudsley National Health Service Trust, UK; **Kristian Arendt**, PhD, Department of Psychology and Behavioural Sciences, Aarhus University, Denmark; **Susan Bögels**, PhD, Research Institute Child Development and Education, University of Amsterdam, The Netherlands; **Peter Cooper**, DPhil, School of Psychology and Clinical Language Sciences, University of Reading, UK, and Department of Psychology, Stellenbosch University, South Africa; **Cathy Creswell**, DClinPsy, PhD, School of Psychology and Clinical Language Sciences, University of Reading, UK; **Tim Dalgleish**, PhD, MRC Cognition & Brain Sciences Unit, Cambridge, UK; **Catharina A. Hartman**, PhD, Department of Psychiatry, University of Groningen, University Medical Center Groningen, The Netherlands; **Einar R. Heiervang**, PhD, Institute of Clinical Medicine, University of Oslo, Norway; **Katrin Hötzel**, PhD, Department of Psychology, Ruhr-Universität Bochum, Germany; **Jennifer L. Hudson**, PhD, Centre for Emotional Health, Department of Psychology, Macquarie University, Sydney, Australia; **Tina In-Albon**, PhD, Clinical Child and Adolescent Psychology, Universität Koblenz-Landau, Germany; **Kristen Lavallee**, PhD, Department of Psychology, University of Basel, Switzerland; **Heidi J. Lyneham**, PhD, Centre for Emotional Health, Department of Psychology, Macquarie University, Sydney, Australia; **Carla E. Marin**, PhD, Yale University, Child Study Center, New Haven, Connecticut, USA; **Richard Meiser-Stedman**, PhD, MRC Cognition & Brain Sciences Unit, Cambridge, UK; **Talia Morris**, BPsych, Centre for Emotional Health, Department of Psychology, Macquarie University, Sydney, Australia; **Maaike H. Nauta**, PhD, Department of Clinical Psychology and Experimental Psychopathology, University of Groningen, The Netherlands; **Ronald M. Rapee**, PhD, Centre for Emotional Health, Department of Psychology, Macquarie University, Sydney, Australia; **Silvia Schneider**, PhD, Department of Psychology, Ruhr-Universität Bochum, Germany; **Sophie C. Schneider**, BPsych, Centre for Emotional Health, Department of Psychology, Macquarie University, Sydney, Australia; **Wendy K. Silverman**, PhD, Yale University, Child Study Center, New Haven, Connecticut, USA; **Mikael Thastum**, PhD, Department of Psychology and Behavioural Sciences, Aarhus University, Denmark; **Kerstin Thirlwall**, DClinPsy, **Polly Waite**, DClinPsy, School of Psychology and Clinical Language Sciences, University of Reading, UK; **Gro Janne Wergeland**, MD, Department of Child and Adolescent Psychiatry, Haukeland University Hospital, Bergen, and Anxiety Disorders Research Network, Haukeland University Hospital, Norway; **Gerome Breen**, PhD, King's College London, IoPPN, MRC SGDP Centre, UK, and National Institute for Health Research Biomedical Research Centre, South London and Maudsley National Health Service Trust, UK; **Thalia C. Eley**, PhD, King's College London, IoPPN, MRC SGDP Centre, UK

## Abstract

**Background**

Anxiety disorders are common, and cognitive–behavioural therapy (CBT) is a first-line treatment. Candidate gene studies have suggested a genetic basis to treatment response, but findings have been inconsistent.

**Aims**

To perform the first genome-wide association study (GWAS) of psychological treatment response in children with anxiety disorders (*n* = 980).

**Method**

Presence and severity of anxiety was assessed using semi-structured interview at baseline, on completion of treatment (post-treatment), and 3 to 12 months after treatment completion (follow-up). DNA was genotyped using the Illumina Human Core Exome-12v1.0 array. Linear mixed models were used to test associations between genetic variants and response (change in symptom severity) immediately post-treatment and at 6-month follow-up.

**Results**

No variants passed a genome-wide significance threshold (*P* = 5 × 10^−8^) in either analysis. Four variants met criteria for suggestive significance (*P*<5 × 10^−6^) in association with response post-treatment, and three variants in the 6-month follow-up analysis.

**Conclusions**

This is the first genome-wide therapygenetic study. It suggests no common variants of very high effect underlie response to CBT. Future investigations should maximise power to detect single-variant and polygenic effects by using larger, more homogeneous cohorts.

Anxiety disorders are the most common psychiatric disorders, with a lifetime prevalence of ~30%.^1^ They are a major cause of global disability, and impose considerable economic burdens on society.^2,3^ They commonly have their onset in childhood or adolescence and have been linked to the occurrence of later disorders, including depression and conduct disorder.^1,4^ Adults with anxiety disorders show rates of childhood anxiety diagnoses significantly above baseline.^5^ Given this potential gateway effect, and the distress caused by these disorders, there is a need to optimise and understand treatment effectiveness in childhood.

Cognitive–behavioural therapy (CBT) is a first-line treatment for anxiety disorders in the UK, with 59% remission reported immediately post-treatment.^6,7^ Despite this high reported efficacy, variability exists in patient response that may be influenced in part by genetic variants. Multiple studies examining the genetics of differential response to psychological therapies (therapygenetics^8^) have been undertaken, and variants in seven genes (*5HTT/SLC6A4, TPH2, MAOA, COMT, NGF, BDNF* and *GRIK4*) have been implicated at least once in studies of CBT for anxiety disorders.^9^ However, findings have proven difficult to replicate,^10^ and the direction of effects found inconsistent. These problems may result from the low power of small cohort sizes, resulting in a high rate of false positives, and a narrow focus on a few genes that may have limited relevance to the phenotype.

Genome-wide association studies (GWAS) provide a hypothesis-neutral alternative, agnostic to prior assumptions of relevance and with the potential to discover novel findings at a single variant level. By analysing thousands of variants across the genome, GWAS yield more information than the candidate gene approach, allowing for the acknowledgement and control of confounds such as ancestry and the quality of genotyping. Genome-wide information can also be used to investigate associations between phenotypic change and different levels of the genetic architecture, including the effect of all variants in a given gene, and the effect of all genotyped variants across the genome. However, the explicit requirement for multiple testing correction in GWAS imposes a need for large sample sizes.

Although GWAS have not been used to study response to CBT, they have shown early promise in studying anxiety disorders. Genetic influences on the development of anxiety disorders may indicate processes underlying treatment response, and provide interesting genetic candidates.^11^ A detailed review of the genetics of anxiety disorders is available elsewhere.^12^ In brief, one variant, rs7309727 (*TMEM132D)*, was associated with panic disorder in a cohort of European ancestry (*P* = 1.1 × 10^−8^_,_ odds ratio (OR) = 1.45 (95% CI 1.20–1.72).^13^ A variant in the *TMEM16B* gene was reported at genome-wide significance in a Japanese cohort with panic disorder, but was not significant in replication analyses.^14^ Two GWAS of post-traumatic stress disorder (PTSD) have identified variants at genome-wide significance in the *TLL1* gene (rs6812849, *P* = 3.13 × 10^−9^, OR not reported)^15^ and *PRTFDC1* (rs6482463, *P* = 2.04 × 10^−9^, OR = 1.47 (95% CI 1.35–1.59)).^16^ However, these results require replication in larger studies; for example, variants in the *RORA* gene previously implicated in a GWAS of PTSD failed to attain significance in a larger replication effort.^17^ No significant findings from the anxiety literature to date had previously been considered in candidate gene studies.^12^

To our knowledge, this is the first GWAS to examine response to psychological therapy in any disorder, and the first to examine treatment response of any kind in anxiety disorders. Participants were drawn from the Genes for Treatment (GxT) study, an international, multisite investigation of clinical, demographic and genetic predictors of response to CBT for anxiety in childhood and adolescence.^10,18^ Two analyses of association between single nucleotide polymorphisms (SNPs) and response to CBT were conducted, investigating change in symptom severity between baseline and immediately post-treatment (post-treatment), and between baseline and 6 months after treatment cessation (follow-up).

## Method

### Study design and sample

A detailed description of the participants and the treatment programmes from which they were drawn is provided elsewhere (online supplemental material).^18^ In brief, participants provided DNA for the GxT study between 2005 and 2013, at 11 sites across the USA, Australia and Western Europe. Children and adolescents (5–17 years old, 94% aged 5–13) were included if they met DSM-IV criteria^19^ for a primary anxiety disorder diagnosis, with further psychiatric diagnoses made as appropriate. Exclusion criteria were significant physical or intellectual impairment, and the presence of psychotic symptoms. All participants completed a full course of individual-based CBT (with or without parental involvement), group-based CBT or guided self-help either as part of a trial or as treatment as usual within a clinical research department. All treatments were manualised and treatment protocols across all sites were comparable for core elements of CBT including teaching of coping skills, cognitive restructuring, and exposure.

Assessments were made using the Anxiety Disorders Interview Schedule for DSM-IV, Parent and Child Versions (ADIS-IV-C/P),^20^ except at Bochum (Germany) and Basel (Switzerland) where the German equivalent, Kinder-DIPS,^21^ was used. All participants were assessed prior to and immediately after treatment, with further assessments made at 3-, 6- or 12-month follow-up where possible. Output from the ADIS (or equivalent) was converted into Clinical Severity Ratings (CSR) on a scale of 0–8. A diagnosis was made when the child met the diagnostic criteria and received a CSR of 4 or more, usually based on a composite of parent and child report. Diagnoses were made from the ADIS for multiple anxiety disorders, and primary status allocated to the most severe, defined as the highest CSR, with ties resolved by clinical judgement (online Table DS1(b) and (c)).

To minimise differential assessment across sites, raters at Reading (UK), Oxford (UK) and Aarhus (Denmark) all received training in evaluation from the Sydney (Australia) site, and clinicians at Aarhus received additional training in the ADIS from W.K.S., principal investigator of the Florida (USA) site. As such, standardised assessments were made for at least 85% of the analysed sample (for further details see the online supplement).

### Definition of the treatment response phenotype

As in previous analyses of the GxT sample, outcome was assessed across two periods: baseline to post-treatment and baseline to follow-up. Although dichotomised treatment outcomes are often used in clinical decision making in treatment response, a continuous measure of change in severity provides substantially more power for analyses.^22^

Response post-treatment was therefore defined as percentage change in CSR score between baseline and immediately following treatment. Percentage change, rather than absolute change, was used as it has been shown to better reflect clinical ratings of improvement by its successful use in pharmacogenetics GWAS.^23^ For follow-up analyses, a range of time points were available; assessments taken at the 6-month time point were used, as these were the most complete (*n* = 483). Missing data at this time point was imputed using the best linear unbiased estimates from linear mixture models fitted to the GxT data as part of analyses predicting response from clinical variables alone.^18^ The mixture models included the linear and quadratic effects of time as well as gender, age, primary diagnosis, treatment type and the random effects of individual and trial (for a full explanation, see Hudson *et al*^18^). This allowed us to compute response at follow-up as the percentage improvement in CSR score from baseline to 6 months after the end of treatment. Analyses were performed on residual scores generated from a linear regression of the percentage change measure adjusted for baseline severity, age, gender, treatment type, diagnosis and trial.

Both sets of residual scores were created as output variables from our previous study, which found a number of significant non-genetic influences on treatment outcome (online supplement).^18^

### DNA extraction and genotyping

DNA was collected and extracted using standard protocols, from buccal swabs^24^ and saliva kits (OG-500 / PrepitL2P, DNAgenotek, Kanata, Canada). Sample preparation (including concentration and quantification) prior to genotyping is described in the online supplement. Genotyping was performed on Illumina HumanCoreExome-12v1.0 microarrays (Illumina, San Diego, California, USA), using a standard protocol.^25^ Samples were genotyped in two batches, and randomized by site on each microarray.

### Quality control

SNPs were mapped to build version 37/hg19 of the human genome. Initial genotype calls were made with GenCall software (GenomeStudio, Illumina, San Diego, California, USA), reprocessed to remove poorly performing samples, re-clustered, and manually recalled where appropriate. Further recalling, targeted at improving the identification of rare variants (such as the exonic content of the microarray) was performed using ZCall.^26^ Following recalling, the data were transferred to a multinode computing cluster, and quality control was performed following previously published protocols (online supplement).

Quality controlled data were imputed to the December 2013 release of the 1000 Genomes Project reference (for autosomes; March 2012 release for the X chromosome^27^), using the posterior-sampling method in IMPUTE2 with concurrent phasing.^28^ SNPs imputed with an info metric >0.8 and a minor allele frequency (MAF) >1% were considered best-guess genotypes, and converted back to PLINK binary format using GTOOL (Freeman and Marchini, available at www.well.ox.ac.uk/~cfreeman/software/gwas/gtool.html). SNPs with a genotype probability of <0.9 were set as missing, and those present in <98% of the sample were excluded from the analysis.

### Statistical analysis

Two analyses were performed, examining adjusted percentage change in CSR score from baseline to post-treatment, and from baseline to 6-month follow-up, as described above. Principal component analysis (PCA) of the genotype data was performed to attempt to control for population stratification. However, this yielded components that were not sensitive to differences in outcome. This was likely due to the quantitative nature of the phenotype, the fact that multiple covariates were controlled for in constructing the phenotype, and because participants were drawn from a variety of sites across the globe (online supplement). Accordingly, PCA was deemed unsuitable for controlling for population stratification, prompting the adoption of mixed linear modelling for the association analyses (MLMA). MLMA uses genome-wide genotype data to derive a genomic relationship matrix (GRM), which is used to control for genetic similarity between participants as a random effect.^29^

MLMA association analysis was performed in GCTA, using the *mlma-loco* option for autosomes and the *mlma* option for the X chromosome (online supplement).^30^ For each SNP in the study, percentage change in CSR was regressed on the number of copies of the reference allele of the SNP (0, 1 or 2), weighted by its additive effect. A random effect of genetic similarity (from the GRM) was included as a covariate, as were fixed effects of sample concentration at genotyping, sample type (buccal swab or saliva), and ultrafiltration status (whether the sample was filtered in preparation for genotyping; online supplement). Using the assumptions of this approach, power for the GWAS was estimated using the Genetic Power Calculator.^31^ The sample of 980 participants has 80% power to detect a variant explaining ~4% of variance and 1% power to detect variants explaining 1%.

Results from the association analysis were clumped according to *P*-value using PLINK.^32,33^ Each clump is represented by a sentinel SNP (that with the lowest *P*-value in the clump), and contains all SNPs in linkage disequilibrium with the sentinel (*R*^2^>0.25, within 250kb of the sentinel). One imputed sentinel SNP in the 6-month follow-up analysis was on the borderline of genome-wide significance (rs72850669, *P* = 7.54 × 10^−8^), and was re-genotyped *post hoc* (LGC Genomics, Teddington, UK). This showed the genotype calling of rs72850669 was unreliable (data not shown), and it was removed from the analyses.

To assess the ability of the GWAS to replicate previous findings, the association of SNPs implicated in CBT response in previous candidate gene studies was examined.^9^ Exploratory secondary analyses were performed to assess the combined effects of SNPs on response (details can be found in the online supplement). The proportion of variance in CSR change across time accounted for by all the SNPs in the study was assessed with univariate genomic-relatedness-matrix restricted maximum likelihood (GREML), performed in GCTA using the GRM derived for the GWAS. Polygenic risk score profiling was used to investigate the ability of external data-sets to predict CBT response, using risk profiles from publicly available GWAS of major depressive disorder^34^ and schizophrenia,^35^ as well as from a meta-analysis of response to antidepressants.^36^ To test the ability of the GxT data to predict response to CBT, five analyses were performed. Participants with generalised anxiety disorder, separation anxiety, social phobia and specific phobia, and those from the Reading (UK) site, were separately removed from the dataset and risk profiles derived from the remaining participants. Each profile was then used to predict outcome in the relevant set of removed participants.

### Ethics

All trials and collection of samples were approved by site-specific human ethics and biosafety committees. Parents provided informed consent, children provided assent. The storage and analysis of DNA was approved by the King's College London Psychiatry, Nursing and Midwifery Research Ethics Sub-Committee.

## Results

Sample and SNP exclusions are shown in online Fig. DS1. Phenotype and high-quality genotype data were available for 939 participants in the analysis post-treatment, with an additional 41 participants available for analysis at 6-month follow-up (*n* = 980). Baseline demographic information for these 980 participants is described in online Table DS1(a). The position of the samples on principal component axes derived from the HapMap reference populations suggests 92% of the sample are of White Western European ancestry.^37^ A total of 260824 common SNPs passed quality control, which rose to 3017604 SNPs when imputed genotypes were added.

**Fig. 1:**
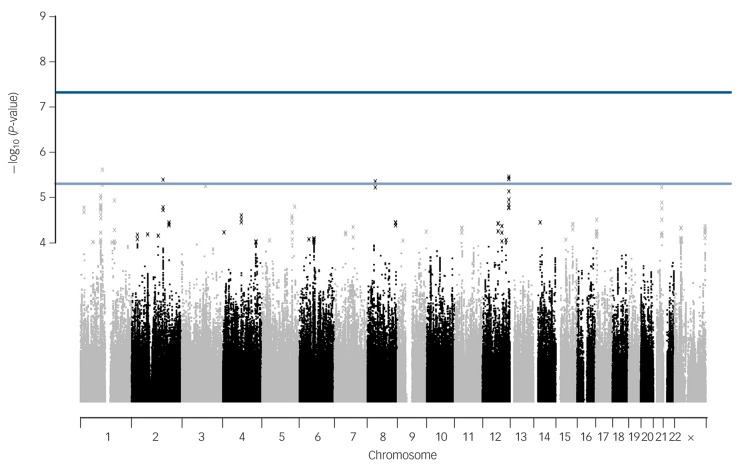
Manhattan plot of genetic associations with cognitive–behavioural therapy response baseline to post-treatment. X-axis shows the top million most associated single nucleotide polymorphisms, arranged by position on the chromosome. Lines show conventional thresholds for genome-wide significance (*P* = 5 × 10^−8^) and suggestive significance (*P* = 5 × 10^−6^).

**Fig. 2:**
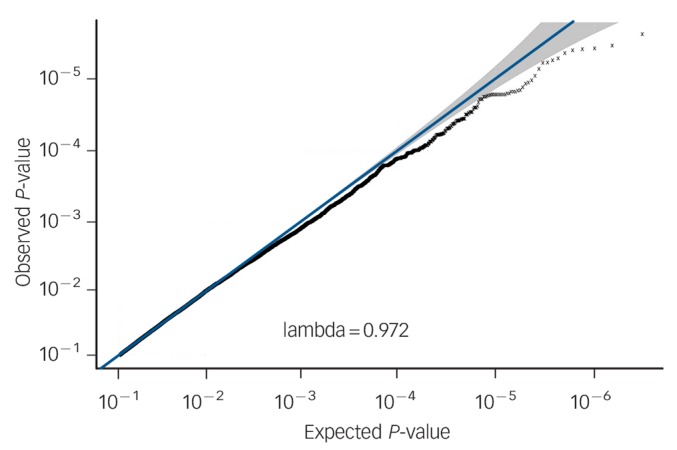
Quantile–quantile plot of *P*-values (pruned for linkage disequilibrium) from genetic associations with cognitive–behavioural therapy response post-treatment. X-axis shows spread of *P*-values expected under the null chi-squared distribution. Y-axis shows observed data. Grey region shows rough 95% confidence intervals around each point on the line x = y. Lambda median is a measure of inflation of the observed distribution of associations compared with expected null distribution. Lambda ⩽1 implies no inflation.

**Fig. 3:**
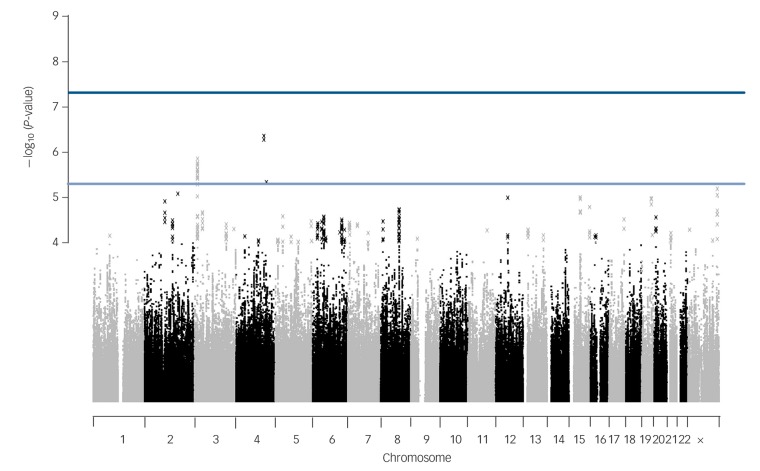
Manhattan plot of genetic associations with cognitive–behavioural therapy response baseline to 6 months after treatment.

**Fig. 4:**
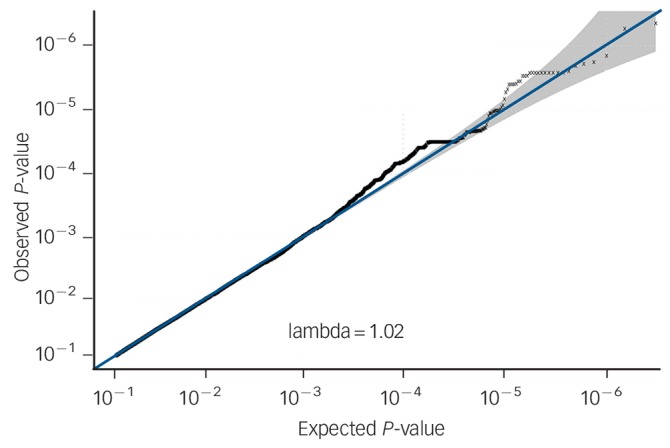
Quantile–quantile plot of *P*-values from genetic associations with cognitive–behavioural therapy response baseline to 6-month follow-up, including lambda median.

No SNPs were found at formal genome-wide significance for either analysis (all *P*>5 × 10^−8^). In the post-treatment analysis, four independent clumps passed threshold for suggestive significance (*P*<5 × 10^−6^; Table 1 and Fig. 1). Quantile–quantile plots show no departure from the chi-squared distribution of *P*-values expected under the null hypothesis, suggesting there is no underlying inflation of association statistics by uncontrolled confounds (lambda median = 0.972, Fig. 2). Three independent clumps were suggestive of significance in the 6-month follow-up analysis (Table 2 and Fig. 3), with no evidence of inflation (lambda = 1.02, Fig. 4). All clumps with *P*<1 × 10^−4^ are displayed in online Table DS2.

**Table 1:** Independent clumps associated with cognitive–behavioural therapy response at post-treatment with *P*<5 × 10^−6^

Sentinel SNP	CHR	Clump BP	Sentinel SNP *P*	Sentinel SNP MAF	Sentinel SNP information	Genes +/−100kb
rs10881475	1	108113663–108203647	2.45 × 10^−6^	0.187	0.993	NTNG1, VAV3

rs11834041	12	128232721–128239057	3.50 × 10^−6^	0.135	Genotyped	–

rs12464559	2	152498699–152679462	4.09 × 10^−6^	0.0410	0.941	NEB, ARL5A, CACNB4

rs881301	8	38322346–38332318	4.46 × 10^−6^	0.403	Genotyped	WHSC1L1, LETM2, FGFR1, C8orf86

SNP, single nucleotide polymorphism; CHR, chromosome; BP, base pair; MAF, minor allele frequency.

**Table 2:** Independent clumps associated with cognitive–behavioural therapy response at 6-month follow-up with *P*<5 × 10^−6^

Sentinel SNP	CHR	Clump BP	Sentinel SNP *P*	Sentinel SNP MAF	Sentinel SNP information	Genes +/−100kb
rs72711240	4	135657189–135695807	4.49 × 10^−7^	0.0269	0.903	–

rs9875578	3	13707416–13810670	1.43 × 10^−6^	0.424	0.994	FBLN2, WNT7A

rs6813264	4	146509970–146631854	4.68 × 10^−6^	0.410	Genotyped	SMAD1, MMAA, C4orf51, ZNF827

SNP, single nucleotide polymorphism; CHR, chromosome; BP, base pair; MAF, minor allele frequency.

A secondary analysis with increased power was performed restricted to nine SNPs previously associated with response to CBT in candidate gene studies (five other SNPs have been previously implicated in CBT response, but did not pass quality control). Assuming a significance threshold of 0.005455 (0.05/9), none of the nine previously associated SNPs was significant (Table 3 and online supplement). The sample had 80% power to detect an SNP accounting for 1.4% of variance at this significance threshold, suggesting any effect of these SNPs in this data-set is smaller than this.

**Table 3:** Genome-wide association study *P*-values of single nucleotide polymorphisms (SNPs) previously associated with cognitive–behavioural therapy response.^12,a^

Gene	SNP	*P* (post-treatment)	*P* (follow-up)
SLC6A4	rs25531	Imputed with info <0.8	Imputed with info <0.8

HTR2A	rs6311	0.4717	0.9692
	rs6313	0.5451	0.8109
	rs6314	Imputed with info < 0.8	Imputed with info <0.8
	rs7997012	Completeness after imputation <0.98	Completeness after imputation <0.98

TPH2	rs4570625	Completeness after imputation <0.98	Completeness after imputation <0.98

COMT	rs4680	0.7699	0.5956

NGF	rs6330	0.5093	0.4559

BDNF	rs6265 (val158met)	0.3408	0.9078
	rs7934165	0.5231	0.9880
	rs1519480	0.8211	0.5013
	rs11030104	0.3158	0.9675

GRIN2B	rs1019385	Imputed with info <0.8	Imputed with info <0.8

GRIK4	rs1954787	0.1315	0.1914

a. No *P*-value is significant after multiple testing correction.

Exploratory secondary analyses (GREML, gene-wide analyses and polygenic risk score profiling) were performed. No significant estimate of SNP heritability could be obtained from GREML, and the effect of adding principal components was minimal. In the post-treatment analysis, all estimates were non-significant. In the 6-month follow-up data the highest estimate was 0.0797 (95% CI −0.194 to 0.35) without principal components. The power of univariate GREML in the sample was estimated for a range of true heritabilities.^38^ Power ranged from 9 to 46% assuming true heritability between 0.2 and 0.6. To achieve 80% power within this range of heritabilities will require 1450–4450 samples (for heritabilities between 0.6 and 0.2).

Polygenic risk score profiling failed to generate predictions that were consistently significant, either for external GWAS or in the internal predictions of response.

## Discussion

### Main findings

We report the first genome-wide association study of psychological therapy. Although no region reached genome-wide significance, the single SNP and polygenic results are consistent with the wider literature of treatment genetics in psychiatry, given the sample size studied. Genome-wide significant variants detected in GWAS of psychiatric phenotypes have shown small effect sizes (with the exception of late-onset dementia), requiring thousands of participants to discover. The pattern of results in psychiatric genomics to date suggests that a critical number of participants (varying by disorder) are required before robust findings begin to be made. In studies of schizophrenia, this critical number was ~9000 cases.^39^ Our results, although preliminary, suggest response to CBT could be a complex phenotype at the early point of this trajectory, although the critical sample size is not yet clear.

The purpose of this study was to identify genetic variants capable of predicting change in symptom severity during treatment. No common, high-effect SNPs were identified, suggesting that it is very unlikely a single variant could be used as a predictor. This also places an upper bound on expected effect sizes in studies of CBT response. This is relevant considering that neither GWAS replicated previous findings from the literature. This does not appear to be due to insufficient statistical power. For example, the *COMT* val158met polymorphism (rs6265) was reported to account for 8% of variance in CBT response in adults with panic disorder, well above the 4% of variance explained for which this GWAS was powered.^40^ Failure to replicate previous findings from the candidate gene literature has proved common in psychiatric genetics, whereas GWAS is proving more reliable.^35,41^ The failure to replicate any published variants suggests previous assumptions of gene relevance may be erroneous, resulting from underpowered candidate gene studies that overestimated the likely effect sizes of studied variants, and that reported variants are likely to be false positives, or to have effect sizes inflated due to winner's curse.^42^ Proximity to a gene does not imply an effect on gene expression, so the failure to replicate the effects of candidate SNPs does not exclude a role for candidate genes, as the SNPs assessed may not capture true functional variation.

Not all candidate variants are SNPs, and one limitation of GWAS is the difficulty of assessing structural variants not captured by the probes on microarrays. For example, we cannot comment on the previously reported role of the *MAOA*-u variable number tandem repeat in CBT response.^43^ Nor could we assess the effect of the *5HTTLPR* variant of *SLC6A4*, previously associated with remission from anxiety disorders at follow-up; however, we directly genotyped this variant in this cohort, and were unable to replicate our earlier finding.^8,10^

Although small when compared with high-profile studies such as the PGC studies in schizophrenia and depression,^34,35^ our sample is similar in size to studies in the depression pharmacogenetic literature.^23,44^ The first of these used a multistage design (*n* = 1532) and identified several associations at nominal significance, but none remained significant after correction for multiple testing.^44^ The second (*n* = 706) found one genome-wide significant locus (for response to nortryptiline treatment) and six loci at suggestive significance across four subanalyses.^23^ More recent meta-analyses were unable to find genome-wide significant variants.^36^ However, a significant GREML estimate of SNP-chip heritability of 42% (95% CI 6%–78%) was identified, suggesting useful information about treatment response can be obtained at the whole-genome level.^45^ Future studies in psychological therapy-genetics should aim to build a cohort of sufficient size to estimate SNP-chip heritability and bivariate genetic correlations, enabling further comparison with pharmacogenetic studies. Such a cohort could act as a target data-set for polygenic risk scoring, exploring the predictive value of variants associated with potentially relevant phenotypes assessed in other GWAS.

### Limitations

There are parallels between the antidepressant GWAS literature and this study, including the necessity of combining many studies to obtain sufficient participants for analysis. Herein, we examined a naturalistic clinical cohort, drawn from CBT trials or from treatment as usual. As all participants received CBT, there was no placebo group for comparison. Therefore, the results may reflect natural regression to the mean, rather than an effect of treatment. Theoretically, a parallel GWAS of change in severity could be performed on wait-list controls to identify associations with regression to the mean. Results from the GWAS of CBT response could be weighted by the likelihood that any given association resulted from regression to the mean. However, this would require deliberate non-treatment of thousands of wait-list controls over a period of at least 7 months for the purpose of comparison only. As CBT is effective in this age group, with significant improvement seen in treated groups relative to wait-list controls, non-treatment would raise serious ethical concerns.^7^ The aim of therapygenetics is to discover predictors of differential response to treatment. These predictors need not capture a treatment effect *per se*; they may describe processes separate to treatment that nonetheless lead to better (or worse) response. Nevertheless, in the absence of a control group, this study specifically examines the association between genetic variation and change in CSR across the period of CBT treatment and follow-up, not the biological mechanism of response to CBT.

The naturalistic nature of the cohort creates heterogeneity, including differences in the details of the treatment given, the target disorder of the treatment, and several participant characteristics. The effectiveness of CBT is influenced by a variety of environmental factors. Some of these can be considered within the design, such as treatment type, diagnosis and severity. Others are less easily accounted for, including therapeutic alliance and other social influences, which may only be partly controlled for by the inclusion of trial as a covariate.^18,46^ This reduces the statistical power of analyses, but should not be viewed as an argument against therapygenetics. The ability to offer personalised advice to patients about treatment could avoid considerable amounts of unnecessary distress and expense. Obtaining a set of genes able to assist in clinical prediction will require a cohort that is powerful enough to detect true variants while remaining clinically representative. Thus, a degree of heterogeneity is unavoidable in studying response to CBT, and similar difficulties in pharmacogenetic GWAS suggest this limitation applies to treatment response genomics more generally.

Combining data from trials at multiple sites necessitated compromises in study design. Participants were included if they completed treatment, but drop-out from treatment is common and likely to be related to poorer response. As such, future studies should aim to include severity data for non-completing participants. This would require appropriate modelling of the treatment period, and the proportion of the treatment process completed, before participation ceased. Similarly, combining measurements from different sites and from participants with varying diagnoses required the use of a general, widely applicable outcome measure. The ADIS fit these requirements well, but relies on clinical judgement derived from parent and child report. It may be less sensitive to the effects of CBT than a self-report measure, and be more vulnerable to site-specific biases. However, a suitable diagnosis-general self-report scale was unavailable, and standardising outcomes to combine multiple diagnosis-specific scales is likely to lead to a generalised and difficult-to-interpret result.

### Future directions

This study represents the first GWAS of psychological therapy. Although no genome-wide significant findings emerged, the spread of significance in the associations captured is similar to other early general psychiatric and pharmacogenetic GWAS. The best approach in the immediate future is to increase the sample size available through combining existing cohorts in mega- and meta-analyses. Such a cohort would allow replication of the findings presented in this paper to be attempted, which currently is not possible due to the lack of an independent cohort of suitable size. However, individual variants are likely to have small effect sizes, so future studies should utilise higher-order approaches such as polygenic risk scoring and GREML to leverage the predictive effects of the whole genome. This would also provide an estimate of heritability, which is difficult to obtain through traditional family-based approaches. If the heritability of CBT response were around 30% (similar to that of anxiety disorders), a high-powered polygenic risk score could capture 10–15% of variance, which could be clinically useful when combined with known environmental risk factors.^47^ However, creating such a score will require a sample size of at least 10 000, which would involve considerable effort to obtain.

Alternative approaches may also yield interesting findings. Response to CBT is a behavioural change following exposure to a positive environment, so epigenetic studies investigating how these exposures influence gene expression via DNA methylation will be informative.^48^ Similarly, it will be useful to examine changes in gene transcript expression across treatment and in the longer term. Used in parallel to these approaches, studying specific genetic variants remains a potential method of predicting response to CBT (and understanding its biological basis) and genome-wide investigations represent the most promising avenue in which to focus the gathering momentum of therapygenetics.^49^
